# The Aβ Containing Brain Extracts Having Different Effects in Alzheimer’s Disease Transgenic *Caenorhabditis elegans* and Mice

**DOI:** 10.3389/fnagi.2018.00208

**Published:** 2018-07-31

**Authors:** Yufang Yang, Mo Wang, Ping Yang, Zishan Wang, Li Huang, Jing Xu, Wei Wang, Mei Yu, Liping Bu, Jian Fei, Fang Huang

**Affiliations:** ^1^Jing’an District Centre Hospital of Shanghai, State Key Laboratory of Medical Neurobiology and Institutes of Brain Science, Department of Translational Neuroscience, Fudan University, Shanghai, China; ^2^Biomodel Shanghai Research Center for Model Organisms, Shanghai, China; ^3^School of Life Science and Technology, College of Life Sciences, Tongji University, Shanghai, China; ^4^Department of Neurology, Tongde Hospital of Zhejiang Province, Hangzhou, China; ^5^Department of Cardiology and Shanghai Institute of Cardiovascular Diseases, Zhongshan Hospital, Fudan University, Shanghai, China

**Keywords:** Alzheimer’s disease, β-amyloid, transgenic AD *C. elegans*, APP/PS1 transgenic mice, oral administration, RNA-sequencing (RNA-seq)

## Abstract

**Background**: The deposition of β-sheet rich amyloid in senile plaques is a pathological hallmark of Alzheimer’s disease (AD), which is thought to cause neuronal dysfunction. Previous studies have strongly implicated that intracerebral infusion of brain extract containing aggregated β-amyloid (Aβ) is able to induce cerebral amyloidosis thus causing neuronal damage and clinical abnormalities in rodents and nonhuman primates, which are reminiscent of a prion-like mechanism. Prion disease has been documented in cases of prion-contaminated food consumption.

**Methods**: We investigated whether cerebral transmission of Aβ was possible via oral administration of Aβ-rich brain extract in non-susceptible and susceptible host mice by immunohistochemistry, western blotting and behavior tests. Also brain extracts were supplied to AD transgenic *Caenorhabditis elegans*, and paralysis curve were conducted, following detection of Aβ amyloid. RNA sequencing of nematodes was applied then inhibitors for relevant dysregulated genes were used in the paralysis induction.

**Results**: The oral treatment of AD brain extract or normal brain extract neither aggravated nor mitigated the Aβ load, glial activation or the abnormal behaviors in recipient Amyloid precursor protein/presenilin 1 (APP/PS1) mice. Whereas, a significant improvement of AD pathology was detected in worms treated with Aβ-rich or normal brain extracts, which was attributable to the heat-sensitive components of brain extracts. Transcriptome sequencing of CL4176 nematodes suggested that brain extracts could delay worm paralysis through multiple pathways, including ubiquitin mediated proteolysis and Transforming growth factor β (TGF-β) signaling pathway. Inhibitors of the ubiquitin proteasome system and the TGF-β signaling pathway significantly blocked the suppressive effects of brain extracts on worm paralysis.

**Conclusions**: Our results suggest that systemic transmissible mechanisms of prion proteopathy may not apply to β amyloid, at least in terms of oral administration. However, brain extracts strongly ameliorated AD pathology in AD transgenic nematodes partially through TGF-β signaling pathway and ubiquitin mediated proteolysis, which indicated that some natural endogenous components in the mammalian tissues could resist Aβ toxicity.

## Introduction

A number of neurodegenerative diseases, including Alzheimer’s disease (AD), Parkinson’s disease, Huntington’s disease and the prion diseases (also called Creutzfeldt-Jakob disease (CJD)), are characterized by the aberrant aggregation of specific proteins and referred as the proteopathies (Selkoe, 2003). It is well known that prion diseases can be induced by conformationally variant prion from contaminated food or iatrogenic transmission. Many studies have proven that intracerebral infusion of brain extract from AD patients or AD transgenic mice can induce cerebral β-amyloidosis in AD transgenic mice (Kane et al., 2000; Meyer-Luehmann et al., 2006; Hamaguchi et al., 2012; Heilbronner et al., 2013) and nonhuman primates (Ridley et al., 2006), which was reminiscent with the prion transmission. Eisele et al. (2010) further showed that intraperitoneally applied Aβ-containing inoculates induced cerebral β-amyloidosis in Amyloid precursor protein carrying the Swedish mutation (APP23) transgenic mice, whereas 500 μL of AD brain extract (10% wt/vol in Phosphate buffer saline, PBS) administered orally daily for 5 days failed to induce cerebral β-amyloidosis in APP23 transgenic hosts (Eisele et al., 2009), which came into conflict. Recently, Jaunmuktane et al. (2015) demonstrated that the Aβ deposition in the gray matter and the blood vessel walls was found in a certain portion of individuals with iatrogenic CJD, indicating interpersonal transmission of Aβ pathology and cerebral amyloid angiopathy. Therefore, whether food contaminated with Aβ seeds triggers the instigation and spread of Aβ lesions in brain is not completely clear. In this study, AD transgenic *C. elegans* and mice were treated with the Aβ containing brain extracts. Aβ-induced paralysis, overall growth and lifespan were accessed in worms. Moreover, short-term and long-term effects of brain extract feeding on APP processing pathway, Aβ load, neuroinflammation and behaviors in mice were analyzed. In addition, serums from AD patients and normal subjects were treated to AD worms; the effects on Aβ-induced paralysis were also determined.

## Materials and Methods

### Drugs and the Treatments

Thioflavin-S was obtained from Sigma-Aldrich (T1892). The inhibitor of ubiquitin proteasome system, MG-132 (S, R, S), the inhibitor of Transforming growth factor β (TGF-β) signaling pathway, SB431542 and the inhibitor of mammalian target of rapamycin (mTOR) signaling pathway, rapamycin (Sirolimus) were all purchased from Selleck (Houston, TX, USA). MG-132, SB431542 and rapamycin were all dissolved in Dimethyl sulfoxide (DMSO) and added to the medium 1 h before the treatment of brain extracts. Corresponding with the DMSO content of each treatment, DMSO at a final concentration of 0.5% was added in the control groups. The primary antibodies were: mouse anti-Aβ_1–16_ (6E10; SIG-39320, Covance, Princeton, NJ, USA), rabbit anti-APP (Ab32136, Abcam, UK), rabbit anti-APP C-terminal fragments (CTF; A8717, Sigma-Aldrich, St. Louis, MO, USA), rabbit anti-β secretase (anti-BACE1; 5606S, Cell Signal, Danvers, MA, USA), mouse anti-β-actin (C4, Santa Cruz, Dallas, TX, USA), rabbit anti-glial fibrillary acidic protein (anti-GFAP; AB5804, Millipore, USA), mouse anti-GFAP (3670S, Cell Signal, Danvers, MA, USA), rabbit anti-ionized calcium binding adapter molecule 1 (anti-Iba1; 019-19741, Wako, Japan), mouse anti-t-Tau (Tau-5, Millipore, USA) and rabbit anti-p-Tau (pSer202, Life-Span BioSciences, San Jose, CA, USA).

### *C. elegans* Strains

The employed *C. elegans* strains included: the wild-type (WT) strain N2; transgenic strains CL4176, dvIs27 [pAF29(myo-3/A-Beta 1–42/let UTR) + pRF4(rol-6(su1006))]; CL2006, dvIs2 [pCL12 (unc-54/human A beta peptide 1–42) + pRF4]. All nematode strains and *E. coli* strain OP50 were purchased from the *Caenorhabditis* Genetic Center (University of Minnesota, MN, USA). All worm strains were maintained at 20°C except CL4176 maintained at 16°C on solid nematode growth medium (NGM) plated with live OP50 as a food source (Brenner, 1974).

### Mice

Amyloid precursor protein/presenilin 1 (APP/PS1) mice (B6C3-Tg (APPswe, PSEN1-dE9)85Dbo, The Jackson Laboratory, Bar Harbor, ME, USA) and WT littermates were housed in a 12 h light/dark cycle with water and food *ad libitum*. The use of mice was in accordance with the regulations of the Fudan University Animal Care and Use Committee. The use of human serums were approved by the Human Studies Institutional Review Board, Shanghai Medical College, Fudan University and the Human Studies Institutional Review Board, Tongde Hospital of Zhejiang Province. All surgeries were performed under general anesthesia, and all efforts were made to minimize adverse effects.

### Synchronization

Adult hermaphrodites were transferred onto fresh NGM plates to lay eggs for 2–3 h, and then removed. After hatching, approximately synchronized larvae were used in the experiments.

### Tissue Extracts and Oral Feeding of Extracts

Unless stated otherwise, brain extracts were derived from aged (14-month-old) APP/PS1 transgenic mice and age-matched non-transgenic, control mice. Brain extract and extracts of the peripheral tissues (liver, kidney and spleen) were also prepared from 2-month-old C57BL/6 mice. The whole brain or tissues were homogenized at 10% (wt/vol) in sterile PBS, sonicated for 3 × 5 s, and centrifuged at 3000 *g* for 5 min (Meyer-Luehmann et al., 2006). The supernatant was diluted with PBS to 5% (wt/vol) then aliquoted and frozen. For mice experiments, the 5% extract was used unless stated otherwise. For *C. elegans*, protein concentration of the supernatant was determined by Bicinchoninic acid (BCA) assay and then the supernatant was diluted to a concentration of 0.655 mg/ml defined as 1% for experimental use. Five micromolar caffeine was used as a control in the paralysis assay (Dostal et al., 2010). The recipient WT mice and APP/PS1 mice were divided into three groups; one group was treated with PBS while the other two groups were treated with brain extracts from 14-month-old APP/PS1 mice and age-matched WT mice respectively. PBS and brain extracts were delivered via gavage. Mice at the age of 3 months old or 6 months old were given brain extracts once a day for 14 days. Mice were weighed every day before administration and the dosage of feeding was 0.004 ml/g body weight.

### Paralysis Assay

Synchronized CL4176 eggs were maintained at 16°C until hatching. Twenty-four hours after synchronization, hatched L1 nematodes were transferred to a 48-well plate filled with sterile PBS buffer with or without tissue extracts. After a 24 h incubation at 16°C, L3 transgenic larval nematodes were induced to express Aβ_1–42_ by temperature upshift from 16–25°C as previously described (Link et al., 2003). Sixteen hours after induction (40 h after exposure to extracts), we started observation for worm paralysis and the interval between observations varies from 4 h to16 h. CL2006 nematodes were maintained on solid NGM for 4 days, and then transferred to liquid NGM with or without tissue extracts at 20°C. We observed the paralysis of CL2006 nematodes every other day. *E.coli* (OP50) at a concentration of 1 × 10^9^/ml was added into all culture mediums as a food resource. The worms were scored as “paralyzed” when they failed to move their body with the touch of a platinum loop (Sangha et al., 2012). Each assay was independently repeated for at least two times in two different laboratories, with more than 60 worms per group.

### Growth Assay

After 48 h of induction (88 h after exposure to extracts), CL4176 worms were anesthetized with NaN_3_ (10%), and then picked onto a polylysine-coated glass slide and mounted in glycerin jelly. Worms were photographed under the microscope (Olympus, Tokyo, Japan) and the body length and width were determined by measuring the flat surface area of 30 adult nematodes per group with the matched software (cellSens Standard, Olympus).

### Lifespan and Egg-Laying Assay

Thirty synchronized L1 nematodes were picked into each well of the 48-well plates containing brain extract or sterile PBS supplied with OP50. The day of synchronization was defined as day 0 in the survival curves. CL4176 worms were transferred to a fresh NGM plate at day 5 and N2 worms were transferred at day 3. In the spawning period, each adult worm was transferred to another new plate and all eggs and hatching larvae were counted every day from the first day of egg-laying. When the spawning was over, living worms were counted every other day until all worms were dead. All the lifespan experiments were repeated twice independently.

### RNA-Sequencing and Analysis of RNA-Sequencing Data

Total RNA of worms was extracted using TRIzol reagent (TIANGEN, Beijing, China) according to the manufacturer’s instructions. 1 μg RNA per sample was used to prepare strand-specific RNA-Seq libraries by VAHTS™ mRNA-seq v2 Library Prep Kit for Illumina^®^ (Vazyme, Nanjing, China). Barcoded complementary DNA (cDNA) libraries were sequenced on an Illumina HiSeq X10 to obtain 150-bp pair-end reads at an approximate sequencing depth of 20 million reads per sample. Raw sequence reads were initially processed using FastQC (Babraham Institute, Cambridge, UK) for quality control, and then adapter sequences and poor quality reads (read with a quality score <20 in >10% of bases) were removed using Cutadapter. Quality-filtered reads were then mapped to *Caenorhabiditis elegans* genome (ce10) using STAR, and only uniquely mapped reads were kept. Read counts were calculated using HTSeqcount. Differential expression analysis was conducted with Strand NGS software (Agilent Technologies, Santa Clara, CA, USA), in which DESeq2 was used to quantify transcript reads and obtain Z scores and fold change values for individual genes. Genes with *P* < 0.01, and fold change greater than 2 were selected for further analysis. Differences in integrated read density were visualized against the *Caenorhabiditis elegans* genome by using the Venn diagram and heatmap tools of Strand NGS. KEGG pathway analysis and GO categories were manually curated from results of the Database for Annotation, Visualization and Integrated Discovery (DAVID) functional annotation cluster tool. Gene cluster enrichment was calculated using Gene Set Enrichment Analysis (GSEA; Broad Institute, Cambridge, MA, USA). In DAVID annotation system, EASE Score, a modified Fisher Exact *P*-Value, is adopted to measure the gene-enrichment in annotation terms. The threshold of EASE Score used is 0.05 as default.

### Quantitative Real-Time PCR (qRT-PCR)

Total RNA was isolated from *Caenorhabiditis elegans* tissues with TRIzol reagent (TIANGEN, Beijing, China). Next, cDNA was synthesized with reverse transcription kit (TIANGEN, Beijing, China) following the manufacturer’s instructions, quantitative real time polymerase chain reaction (qRT-PCR) was performed on the quantitative thermal cycler (Mastercycler ep realplex, Eppendorf, Germany) using SYBR Green agent (TIANGEN, Beijing, China). Primers used to examine the mRNA expression level of Aβ_1–42_ were 5′-GACGCGGATGCAGAATTCCGA-3′ and 5′-CGCTATGACAACACCGCCCAC-3′. Primers used for qRT-PCR assay to verify the expression profile by RNA-sequencing of *C. elegans* are listed in the Supplementary Table S1.

### Serum of AD Patients and Normal Subjects

Serum was collected during 8:00–9:00 a.m. and stored at −80°C until use. Patients with AD were recruited from Tongde Hospital of Zhejiang Province. Patients were diagnosed of probable AD based on comprehensive evaluation by two experienced subspecialty cognitive neurologists according to NINCDS-ADRDA Alzheimer’s criteria (McKhann et al., 1984) and their revision (Dubois et al., 2007). Exclusion criteria for the AD included metabolic diseases, large vessel strokes, head injuries, severe psychiatric illness and neuro-developmental conditions. All aspects of the study were approved by the Human Studies Institutional Review Board, Shanghai Medical College, Fudan University and the Human Studies Institutional Review Board, Tongde Hospital of Zhejiang Province. The written informed consent was obtained from the patients for the collection of the human samples. The information of AD patients and normal subjects was listed in Supplementary Table S2.

### Tissue Collection

Worms were washed with PBS buffer for three times and collected. Mice were anesthetized with 10% chloral hydrate (3 ml/kg i.p.) and perfused transcardially with physiological saline. For histochemistry analysis, the brains were carefully removed and post-fixed overnight in 4% paraformaldehyde at 4°C. For western blot experiments, the brains were quickly removed; the cortex and hippocampus were dissected on ice for protein preparation.

### Immunofluorescence Staining

Worms were washed with PBS buffer then fixed in 4% paraformaldehyde in 0.1 M phosphate buffer (pH 7.2) for 24 h. After fixation, worms were rinsed twice with 1 ml of 10 mM Tris-HCl (pH 7.5) and then permeabilized by 24 h exposure to β-mercaptoethanol (5% β-mercaptoethanol, 125 mM Tris-HCl, pH 7.4, 1% Triton X-100) at 37 °C followed by collagenase treatment (2 mg/ml for 1–1.5 h at 37 °C) to allow for digestion of the cuticle. Amyloid-β peptide were detected with anti-6E10 monoclonal primary antibody, with AlexaFluor 594 donkey anti-mouse IgG (Molecular Probes) as secondary antibody. The specimens were imaged using Leica confocal microscope (×100 objective, TCSSP-2, Leica, Germany). For mouse brain, it was carried out according to a previous method (Hong et al., 2014). Briefly, mouse brains were fixed in 4% paraformaldehyde in 0.1 M phosphate buffer (pH 7.2) overnight then soaked in 20% sucrose solution for 24 h and stored in 30% sucrose solution for 24–48 h at 4°C. Brains were cut into 30 μm sections with a freezing microtome (Leica, Germany). Brain sections were permeabilized and blocked in 0.01 M PBS containing 10% goat serum and 0.5% Triton X-100 at 37°C for 1 h and then incubated at 37°C for 2 h and 4°C overnight with primary antibodies. Sections were then incubated with Alexa Fluor 488 conjugated donkey anti-mouse and Alexa Fluor 594 conjugated donkey anti-rabbit secondary antibodies (1:1000; Invitrogen, Carlsbad, CA, USA) at 37°C for 1 h. Then the sections were mounted with AQUA-MOUNT™ medium (Thermo Scientific, Waltham, MA, USA) and images were obtained with a Nikon Eclipse microscope (Nikon, Japan).

### Protein Extraction and Western Blot Analysis

The methods for protein extraction and western blot analysis has been previously described (Bian et al., 2008). Briefly, tissues were lysed in RIPA buffer (50 mM Tris-HCl, pH 7.5; 150 mM NaCl; 1% NP-40; 0.5% sodium deoxycholate; 0.1% sodium dodecyl sulfate) containing a protease inhibitor cocktail (Thermo Scientific, Waltham, MA, USA) and sonicated 5 s for five times. After a centrifugation of 15 min at 13,000 rpm, for *C. elegans*, the supernatants were taken and mixed with Pierce™ Lane Marker Reducing Sample Buffer (Thermo Fisher, Waltham, MA, USA) then boiled for 5 min. Protein samples were separated on a 4%–16% tricine-sodium dodecyl sulfate-polyacrylamide gel and blotted onto PVDF membrane. For brain tissues, the supernatants were mixed with SDS-PAGE Sample Loading Buffer (Beyotime, China) then boiled for 5 min. Protein samples were separated on 10% glycine-sodium dodecyl sulfate-polyacrylamide gels and blotted onto PVDF membrane (Immobilon-P; Millipore, USA). Membranes were blocked by 5% non-fat dried milk in TBS-T (10 mM Tris-HCl, 150 mM NaCl and 0.1% Tween, pH 8.0) at room temperature for 2 h and incubated with primary antibodies at room temperature for 2 h. Subsequently, the membranes were washed with TBS-T and incubated with IRDye 800 cw goat anti-rabbit (1:20,000 dilutions in TBS-T, 926–32211, LI-COR, Lincoln, NE, USA) or IRDye 688 cw goat anti-mouse immunoglobulin G (1:20,000 dilutions in TBS-T, 926-32220, LI-COR, Lincoln, NE, USA) and detected by Odyssey infrared imaging system (LI-COR, Lincoln, NE, USA). The protein levels were quantified by densitometry analysis using Quantity One 4.5.2 software (Bio-Rad, Hercules, CA, USA).

### Sandwich ELISA

To quantify levels of soluble and insoluble Aβ_1–42_, the well-established human Aβ42 ELISA kit (KHB3441, Invitrogen, CA, USA) was used. The samples were prepared according to the manufacturer’s protocol. Briefly, the cortex and the hippocampus from 11 month to 12-month old transgenic mice and their WT counterparts were removed from fresh brains and weighed. With the lysate buffer (5 mM guanidine HCl, 50 mM Tris-HCl, pH 8.0), samples were homogenized and centrifuged. Fractions were then analyzed according to the manufacturer’s protocol. Absorbance was determined for each well at 450 nm using a microplate reader (MPM6, Bio-Rad, Hercules, CA, USA).

### Thioflavin-S Staining

Amyloid deposits in tissue sections were visualized with thioflavin-S staining. Briefly, brain sections were rinsed with TBS buffer, then incubated in 0.5% thioflavin-S for 20 min at 37°C and then washed in 50% ethanol for four times every 5 min. Sections were rinsed in TBS and mounted in glycerin jelly. Images were captured under a fluorescence microscopy (Olympus, Japan).

### Open-Field Test

Spontaneous behaviors of mice were tested in open-field test as described previously (Prut and Belzung, 2003). Tests were performed using an automatic-recording open-field working station (MED Associates, Georgia, VT, USA). The open-field box was 29 cm in width, 50 cm in height with white surrounding walls and blue bottom. The whole box was hidden in a light-free chest during the test. Mice were transferred to the behavioral room to adapt to the environment for at least 30 min before test. A mouse was then positioned in the center of the square box. After release, the behavior of the animal was observed and recorded by a video camera system for 15 min. The recording software determined the total walking distance, time of ambulatory movements and resting. Values for the central and residual part of the box were calculated separately (Kalueff and Tuohimaa, 2004).

### Morris Water Maze Test

The Morris water maze test was performed as previously described (Morris, 1984; Vorhees and Williams, 2006). The water maze consisted of a circular pool (120 cm in diameter, 62.5 cm in height) with a white inner surface. The escape platform (10 cm in diameter) was fixed at the position equidistant away from the center and the wall of the tank and submerged 1 cm below the surface. The tank was located in a test room surrounded with various visual cues on the wall. The behavioral-tracking software (Anymaze, Stoelting Co., Wood Dale, IL, USA) recorded the swimming activities of the animal to the platform within 60 s by video tracking. When reaching the platform, the animal was allowed staying for 30 s. If an animal failed to find the platform within 60 s then it was gently guided to the platform and allowed to stay there for 30 s. Each mouse performed over 7 days four trials daily at randomly chosen starting points as a previous protocol (Vorhees and Williams, 2006). Probe sessions were performed 24 h after the last trial. The probe test included a single probe trial in which the platform was removed from the tank and each mouse was allowed to swim for 60 s in the maze from the furthest starting point to the platform.

### Statistical Analysis

All values were shown as the means ± SEM. Statistical analyses were performed using the GraphPad Prism 5.0 software. Paralysis curves of *Caenorhabditis elegans* were analyzed using a paired log rank survival test (Peto and Peto, 1972). For other figures, means between two groups were compared by the two-tailed unpaired Student’s *t*-test (as indicated in the figure legends). Data from multiple groups were analyzed by one-way ANOVA, and two-way ANOVA, followed by the appropriate *post hoc* tests (as indicated in the figure legends) when necessary. *P* ≤ 0.05 was considered statistically significant.

## Results

### Brain Extracts From Both Wild Type and AD Transgenic Mice Delay Paralysis Induced by Aβ Expression in Worms

**Figure 1:**
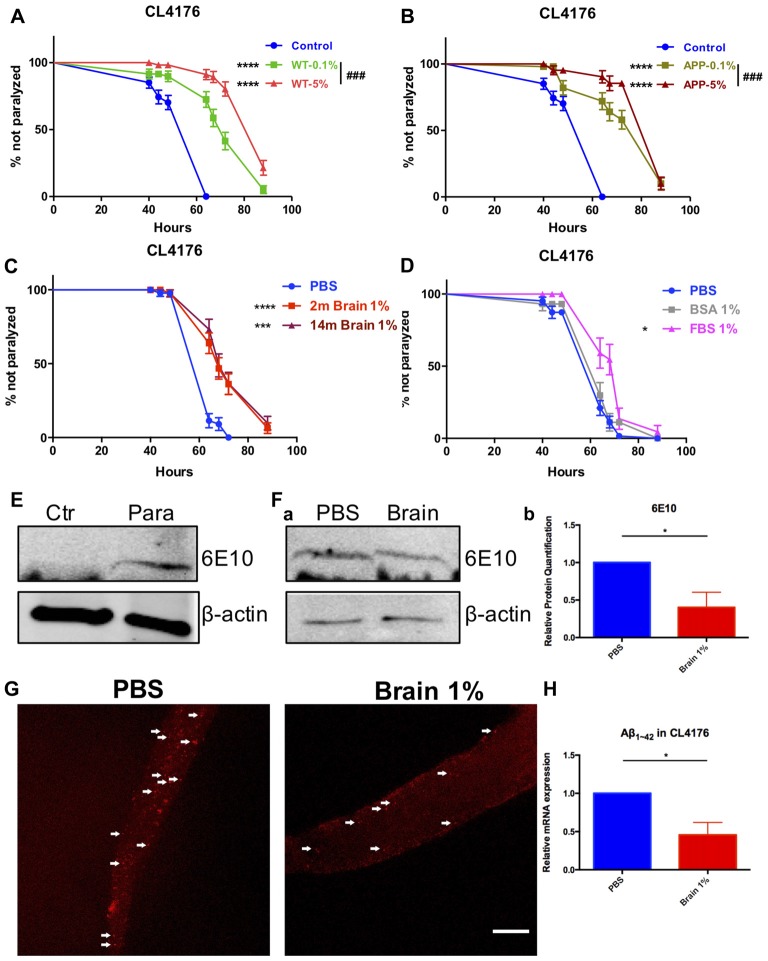
Beta-amyloid (Aβ)-induced paralysis was delayed in Alzheimer’s disease (AD) transgenic nematode CL4176 treated with brain extracts. **(A)** The effect of feeding brain extracts from wild type (WT) mice at 0.1% and 5% concentration. **(B)** The effect of feeding brain extracts from AD transgenic mice at 0.1% and 5% concentration.** (C)** Brain extracts from 2-month-old mice and 14-month-old mice showing similar anti-paralysis effects in CL4176 worms. **(D)** Different effects between 1% fetal bovine serum (FBS) and 1% bovine serum albumin (BSA) on paralysis. Data were analyzed using a paired log rank survival test. Sixty worms were counted per group. The level of significance was shown in brackets (*P < 0.05, ***P < 0.001, ****P < 0.0001, ^###^*P* < 0.001). **(E)** Protein levels of toxic Aβ_1–42_ detected by western blot with antibody 6E10 in CL4176 worms. Transgenic worms were harvested at 40 h with or without temperature induction. **(F)** The effects of 1% brain extracts on Aβ expression. **(a)** Worms were harvested at 64 h after giving extracts and applied to western blot analysis. **(b)** Statistics of Aβ_1–42_ expression levels. Samples were collected from three independent experiments. β-actin served as the internal control. Data were analyzed by unpaired Student’s *t* test. The level of significance was shown in brackets (*P < 0.05). **(G)** Representative fluorescence images for Aβ_1–42_ in CL4176 worms. Worms were collected at 64 h after giving extracts. The toxic Aβ aggregates, stained by 6E10 (red) and indicated with white arrows, were significantly decreased in worms treated with brain extracts. Scale bar: 10 μm. **(H)** The relative mRNA expression level of Aβ_1–42_ in CL4176 worms with or without treating brain extracts. Worms were harvested at 64 h after giving extracts and collected from three independent experiments. Data were analyzed by unpaired *t*-test with equal SD. The level of significance was shown in brackets (*P < 0.05), *n* = 3.

Synchronized eggs of transgenic strain CL4176 were growing at the permissive temperature of 16°C for 24 h. Worms were picked into the liquid medium supplied with brain extracts from WT or AD transgenic mice (14-month-old) of the final concentration of 5%, 1% and 0.1%, respectively and cultured for another 24 h. Then the nematodes were moved to the non-permissive temperature of 25°C. Sixteen hours later, we started to observe the paralysis of worms. We found that brain extracts markedly delayed the onset of paralysis in worms expressing human Aβ_1–42_ and the protective effect of brain extracts were dose dependent, with 5% brain extracts showing better effects compared with 0.1% brain extracts (*P <* 0.0001 and *P =* 0.039 for WT and AD transgenic mouse brain extracts, respectively; Figures 1A,B). There was no difference between the effects of WT and AD transgenic mouse brain extracts at both concentrations (0.1% and 5%; Supplementary Figure S1). Noticeably, the anti-paralysis effect of brain extracts from 2-month-old WT mice were quite close to those from 14-month-old mice at 1% concentration (Figure 1C), thus 1% brain extracts from 2-month-old mice were used in the following experiments. Suppression of paralysis was not observed in CL4176 worms treated with 1% bovine serum albumin (BSA), however worm paralysis was delayed by the treatment of 1% fetal bovine serum (FBS; Figure 1D). 0.01%, 0.1% and 5% of BSA and FBS were further tested in worms. FBS suppressed paralysis of worms at all concentrations while BSA just showed protective effects at the lowest 0.01% concentration (Supplementary Figure S2).

Sixty-four hours after supplying 1% brain extracts (40 h after temperature upshift), CL4176 worms were collected and lysed for western blot to detect toxic Aβ. L3 lavae CL4176 nematodes growing on the NGM plated with or without a 48-h induction by temperature shift were served as control. Aβ could only be detected in temperature-induced transgenic CL4176 nematodes while none of Aβ was produced in worms without induction (Figure 1E). Aβ was both expressed in CL4176 nematodes with or without brain extracts supply (Figure 1Fa). However, the expression of Aβ was significantly reduced in brain extracts-treated worms (Figure 1Fb). Also, by immunofluorescence staining, the Aβ aggregates in CL4176 worms treated with brain extracts were dramatically decreased (Figure 1G). Moreover, by quantitative RT-PCR assay, we found that the mRNA expression level of Aβ_1–42_ was significantly decreased in CL4176 nematodes with the treatment of brain extracts (Figure 1H).

**Figure 2:**
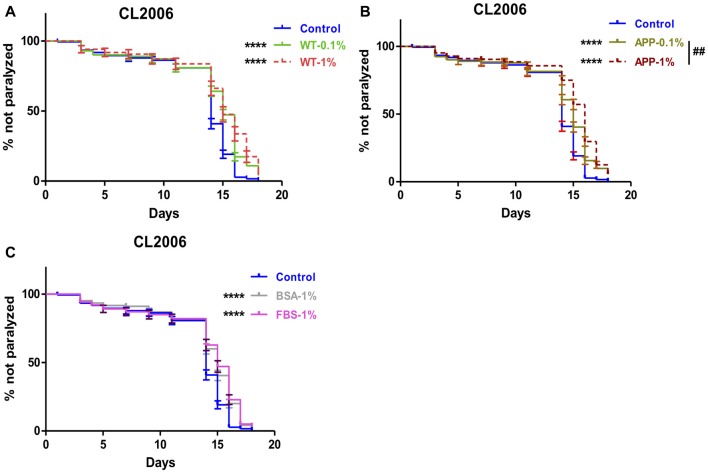
Aβ-induced paralysis was delayed in AD transgenic nematode CL2006 treated with brain extracts. **(A)** The effect of feeding brain extracts from WT mice at 0.1% and 1% concentration. **(B)** The effect of feeding brain extracts from AD transgenic mice at 0.1% and 1% concentration. **(C)** The effects of feeding 1% BSA and 1% FBS. Data were analyzed using a paired log rank survival test. The level of significance was shown in brackets (****P < 0.0001, ^##^*P* < 0.01).

The protective effects of brain extracts were also examined on AD transgenic strain CL2006, in which human Aβ_1–42_ is constitutively expressed. 1% or 0.1% brain extracts were tested in CL2006 nematodes. Numbers of paralyzed worms were counted every 2 days. We found that brain extracts from two genotypes of mice at both 0.1% and 1% concentration significantly delayed the onset of worm paralysis, while brain extracts from AD transgenic mice manifested a dose effect that 1% extracts had better effects than 0.1% brain extracts (*P* = 0.0082; Figures 2A,B). The protective effects of brain extracts from both genotypes of mice were comparable between 0.1% and 1% concentration (Supplementary Figures S3A,B). Suppression of paralysis was also observed with 1% FBS and 1% BSA (Figure 2C). 1% BSA was less effective compared to 1% WT brain extracts (*P* = 0.018) and 1% AD brain extracts (*P* = 0.0012); while 1% FBS showed less effects than 1% AD brain extract (*P* = 0.0311) yet no difference compared to 1% WT brain extract (Supplementary Figures S3C–F).

### Extracts From Mouse Peripheral Tissues Also Delay Paralysis Induced by Aβ Expression in Worms

**Figure 3:**
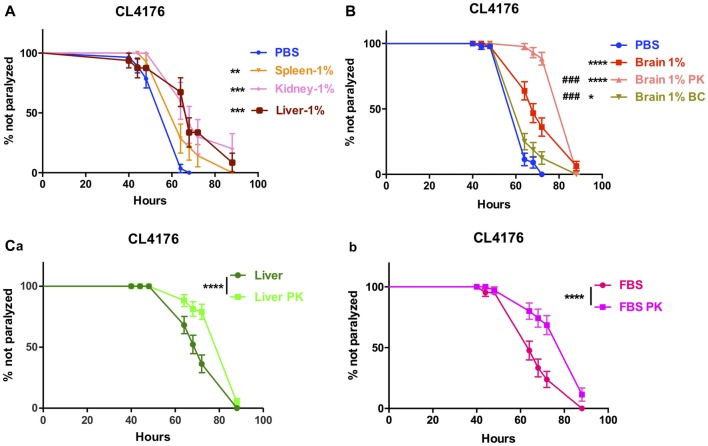
The effects of brain extracts, peripheral tissue extracts and FBS with extra treatment in CL4176. **(A)** The effects of extracts from spleen, kidney and liver on paralysis at 1% concentration. **(B)** The compromised effect of brain extracts after boiling plus centrifugation and the enhanced protective effects of brain extracts after PK plus boiling treatment. **(C)** The enhanced protective effects of liver extracts** (a)** and FBS **(b)** after PK treatment. Data were analyzed using a paired log rank survival test. *P < 0.05, **P < 0.01, ***P < 0.001, ****P < 0.0001, ^###^*P* < 0.001.

The effect of peripheral tissue extracts on the onset of paralysis of CL4176 worms were also determined. The extracts of spleen, kidney and liver all had protective effects at 1% concentration (Figure 3A).

### Protein Components Inside the Brain Extracts Suppress the Paralysis of CL4176 Worms

To further clarify the definite component(s) for suppression of paralysis, first we did the comparison between normal saline-perfused and non-perfused brain and peripheral tissue extracts in the nematode CL4176 and found that the protective effects were similar to each other (Data not shown), thus excluding the affection of blood. Next, we removed proteins inside the brain extracts by boiling and centrifugation (BC treatment) then repeated the experiment in CL4176 transgenic worms. 85.1% of protein component in brain extracts was depleted with BC treatment (Supplementary Table S3) and the suppression of worm paralysis by treated brain extracts was markedly compromised (Figure 3B). By treating the extracts with proteinase K and boiling (PK treatment), 55.2% of protein component in brain extracts was expelled (Supplementary Table S3), to our surprise, the protective effects of brain extracts was significantly enhanced compared with original brain extracts (Figure 3B). PK treatment reduced protein components by 46.7% in liver extracts and 21.5% in FBS (Supplementary Table S3). Similarly, PK-treated liver extracts and FBS showed obviously enhanced protective effects (Figures 3Ca,b).

### Brain Extracts Could Delay Worm Paralysis Mainly Through the TGF-β Signaling Pathway and Ubiquitin Mediated Proteolysis

**Figure 4:**
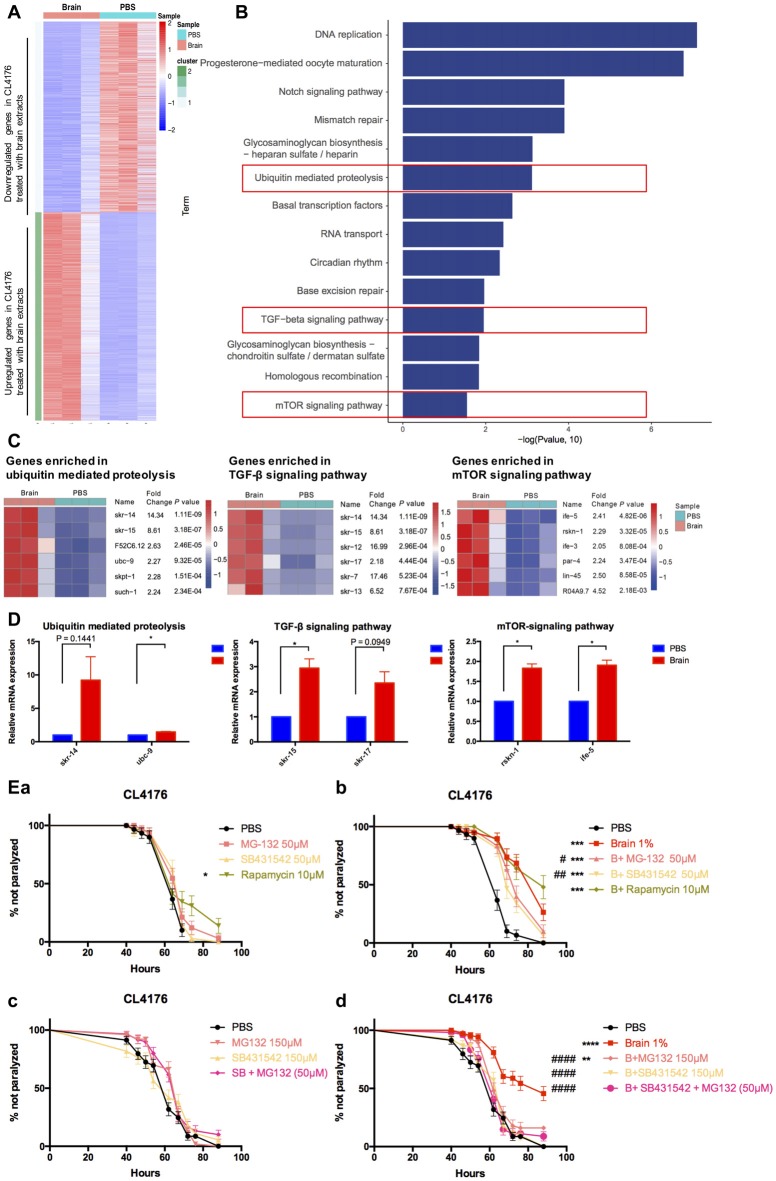
Multiple pathways were involved in the suppressive effects of brain extracts on worm paralysis. **(A)** The heat map of total dysregulated genes in brain extracts-treated CLL4176 worms (*P <* 0.01, fold change ≥2). **(B)** KEGG pathway analysis of genes significantly upregulated in CL4176 treated with brain extracts (*P* < 0.05). **(C)** The heat map showing upregulation of genes that were involved in three KEGG pathways: ubiquitin mediated proteolysis, TGF-β signaling pathway and mammalian target of rapamycin (mTOR) signaling pathway. **(D)** The mRNA expression levels of *skr-14*, *ubc-9*, *skr-15*, *skr-17*, *rskn-1*, *ife-5*, components of the above KEGG pathways, were verified by real time polymerase chain reaction (RT-PCR) with worm collected from another three sets of independent experiments, *P < 0.05, with unpaired *t*-test. **(E)** The effects of brain effects on worm paralysis under the intervention of inhibitors for the three upregulated pathways in CL4176 worms. **(a)** The effects of the inhibitors at low doses on CL4176 worms in Phosphate buffer saline (PBS). **(b)** The effect of brain extracts with the low doses of inhibitors. **(c)** The effects of the inhibitors at high doses on CL4176 worms in PBS. **(d)** The effect of brain extracts with high doses of inhibitors. MG-132: inhibitor of ubiquitin mediated proteolysis; SB431542: inhibitor of Transforming growth factor β (TGF-β) signaling pathway: Rapamycin: inhibitor of mTOR signaling pathway. *P < 0.05, **P < 0.01, ***P < 0.001, ****P < 0.0001, compared with PBS group; ^#^*P* < 0.05, ^##^*P* < 0.01, ^####^*P* < 0.0001, compared with Brain group.

To decipher the transcriptional profiles of CL4176 worms with or without the treatment of brain extracts, we collected worm samples at 64 h after giving 1% brain extracts (40 h after temperature shift) and the control from three independent experiments for RNA sequencing. The result revealed that 1754 (52% of all dysregulated genes) genes were upregulated and 1606 (48% of all) genes were downregulated in brain extracts- treated CL4176 worms compared with the control (Figure 4A, Supplementary Data Sheet 1). KEGG pathway analysis identified several pathways that most significantly upregulated in CL4176 nematodes treated with brain extracts, some of which have been widely reported playing critical roles in AD pathological process, such as the ubiquitin mediated proteolysis, the TGF-β signaling pathway, and the mTOR signaling pathway (Figure 4B). The heat map presented the top six upregulated genes enriched in each of the three pathways (Figure 4C) and some of them were verified by qRT-PCR (Figure 4D) using different sets of worms. *skr-14*, encoding a homolog of Skp1, the core component of the SCF ubiquitin-ligase complex, and *ubc-9*, encoding an E2 ubiquitin-conjugating enzyme, were upreguated in worms with brain extract treatment (*P* = 0.1441 and *P <* 0.05 for *skr-14* and *ubc-9*, repectively). *skr-15* and *skr-17*, both related to Skp1 protein and enriched in TGF-β signaling pathway, were increased by the treatment of brain extracts (*P* = 0.0949 for *skr-15* and *P <* 0.05 for *skr-17*). Both of *rskn-1* and *ife-5* are involved in the mTOR signaling pathway. Higher expression levels were identified in worms treated with the brain extracts (*P <* 0.05 for *rskn-1* and *ife-5*), which was in agreement with the sequencing results. To figure out the key pathway(s) by which the brain extracts militate against worm paralysis, we used relevant inhibitors to suppress the pathways and conducted paralysis assays in CL4176 worms (Figure 4E). MG132, an inhibitor of ubiquitin proteolysis, and SB431542, an inhibitor of TGF-β signaling pathway, both significantly but not completely blocked the effects of brain extracts at the concentration of 50 μM (Figure 4Eb), while none of them affected the worm paralysis progress compared with the PBS control (Figure 4Ea). On the contrary, rapamycin, an inhibitor of mTOR signaling pathway, did not alter the effects of brain extracts (Figure 4Eb) but the drug alone suppressed worm paralysis in PBS at the concentration of 10 μM (Figure 4Ea). Since that MG132 (50 μM) and SB431542 (50 μM) partially attenuated the protective effects of brain extracts, higher doses of the two drugs were applied to worms. The effects of brain extracts were totally abolished by treatment of 150 μM SB431542, also significantly but not completely diminished by treatment of 150 μM MG132 (Figure 4Ed). The combined treatment of the two drugs were also applied to the nematodes at the concentration of 50 μM each. The combined treatment totally abolished the paralysis-delaying effects of brain extracts (Figure 4Ed). All treatments alone in PBS showed no toxicity to the nematodes (Figure 4Ec).

### Brain Extracts Promote Growth, but Have No Overall Effects on Fertility and Lifespan in *C. elegans*

**Figure 5:**
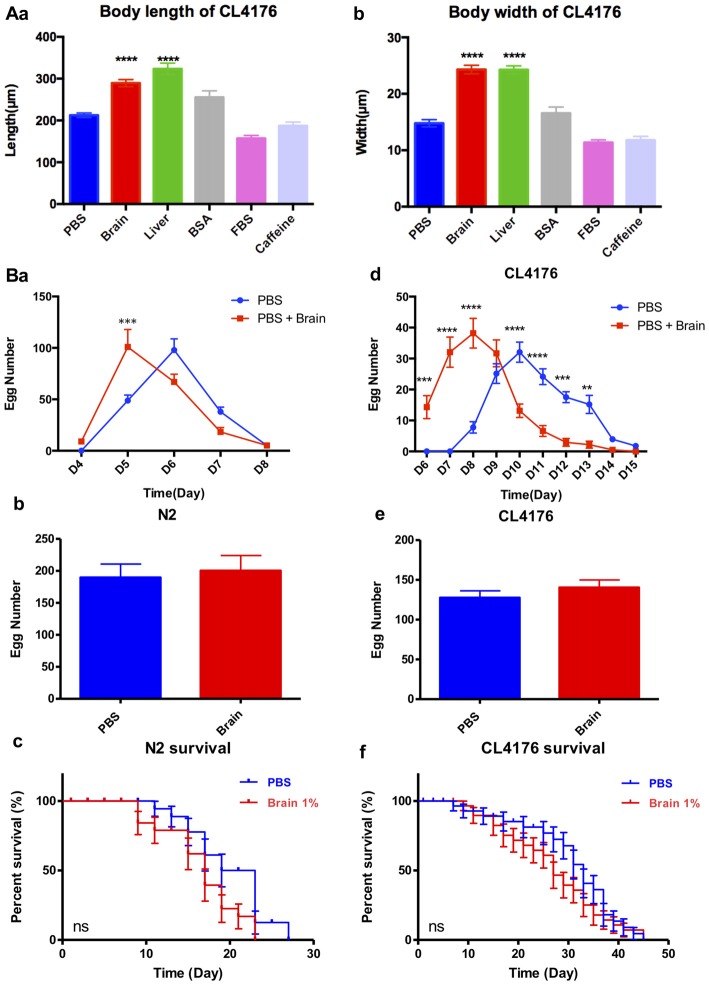
The effect of brain extracts on the growth of CL4176 and WT N2 worms. **(A)** The effect of brain extracts on body sizes in CL4176 worms. **(a)** The body length of worms. **(b)** The body width of worms. Data were analyzed with one-way ANOVA, Tukey’s multiple comparisons *post hoc* that each group was compared with the PBS group. **(B)** The effect of brain extracts on fertility and lifespan of worms. **(a–c)** The egg number in each spawning day, total egg number and life span of N2 worms. **(d–f)** The egg number in each spawning day, total egg number, and life span of AD transgenic CL4176 worms. Egg numbers by days were analyzed with two-way ANOVA, Tukey’s multiple comparisons *post hoc*. Total egg numbers were analyzed with unpaired Student’s *t*-test. Life spans were presented as survival curves and analyzed with a paired log rank test. **P < 0.01; ***P < 0.001, ****P < 0.0001.

The sequencing results showed that brain extracts upregulated the TGF-β signaling pathway and the mTOR signaling pathway. The TGF-β signaling pathway has been reported promoting larval development and extending body size of worms (Gunther et al., 2000; Tewari et al., 2004; Dineen and Gaudet, 2014), while reducing the activity of mTOR would increase lifespan in *C. elegans* (Richardson et al., 2015). We further examined the effects of 1% brain and peripheral tissue extracts on worm growth, fertility and lifespan. CL4176 nematodes were harvested for the measurement of body size at 88 h after feeding the extracts. Both of brain and liver extracts significantly increased the body length and width of worms compared with the control PBS, 1% BSA, 1% FBS or 5 mM caffeine (Figures 5Aa,b). After PK treatment, the growth promoting effects of brain and liver extracts were comparable to their non-treated controls (Supplementary Figure S4). For the assays of fertility and lifespan, brain extracts were tested in WT nematode N2 besides transgenic nematode CL4176. N2 larval1 nematodes were given brain extracts for 2 days then transferred to NGM plates at 20°C and CL4176 larval1 nematodes were given brain extracts for 4 days then transferred to NGM plates at 16°C. Notably, spawning time in both N2 and CL4176 worms treated with brain extracts antedated that of the controls, whereas the total numbers of eggs were not impacted (Figures 5Ba–e). Moreover, the treatment of brain extracts did not affect the lifespan of N2 and CL4176 strains (Figures 5Bc,f).

### Serum From AD Patients and Normal Subjects Has No Difference in the Effects of Anti-paralysis

**Figure 6:**
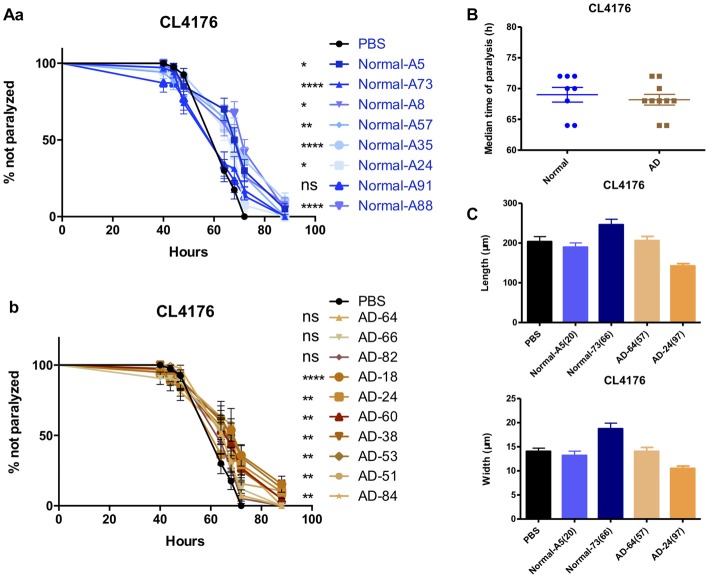
The effects of serums from normal subjects and AD patients on the paralysis in CL4176. **(A)** The effects of serum from normal subjects **(a)** and AD patients **(b)** in CL4176 worms. **(B)** Comparable median time of paralysis in CL4176 worms by feeding serum from normal subjects and AD patients.** (C)** No growth-promoting effects by feeding serum. Paralysis data were analyzed using a paired log rank survival test. Median time of paralysis was analyzed with two-tailed unpaired Student’s *t*-test. Body size was analyzed with One-way ANOVA, Tukey’s multiple comparisons *post hoc*. *P < 0.05; **P < 0.001; ****P < 0.0001, ns: *P* > 0.05, no significant difference.

Enlightened by the anti-paralysis effects of FBS on AD transgenic nematodes, we further tested the effects of serum from AD patients and normal subjects in AD worms. Seventy percent of AD serum (*n* = 10) and 87.5% normal serum (*n* = 8) exhibited the protecting ability in CL4176 worms (Figures 6Aa,b). Serum from AD patients and normal subjects had no difference in the effects of paralysis suppression in view of the median time of paralysis (Figure 6B). However, none of the serum showed any promoting effects on worm growth (Figure 6C).

### Brain Extracts Do Not Alter APP Processing Pathway, Tau Expression, Aβ Load or Glial Activation in the Brains of Recipient Mice

**Figure 7:**
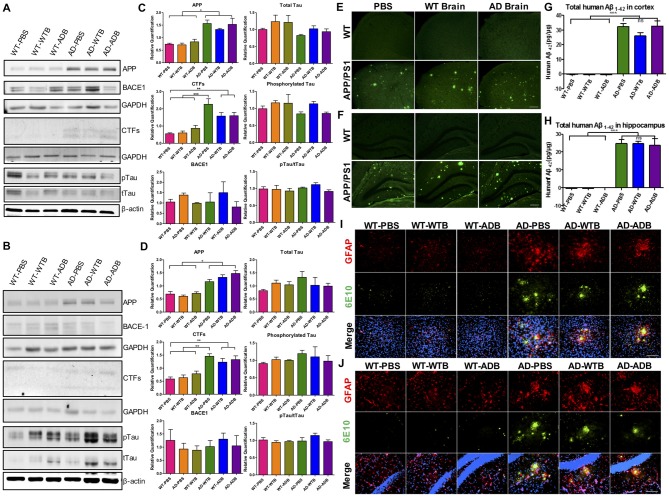
Aβ load and neuroinflammation in 11-month-old mice with gavage. The depicted western blot indicates the expression levels of amyloid precursor protein (APP), β secretase (BACE1), C-terminal fragments (CTFs), total Tau and phosphorylated Tau in the cortex **(A)** and hippocampus **(B)** of 11-month-old mice with different treatments at the age of 6 months old. The relative expression levels of proteins in the cortex **(C)** and hippocampus **(D)** were shown in the diagram (*n* = 3–5 for APP and CTFs and *n* = 3 for BACE1). GAPDH or β-actin served as the internal control. The Aβ load and amyloid plaques in the cortex **(E)** and hippocampus **(F)** of transgenic AD and WT mice were stained with Thioflavin S. The toxic Aβ_1–42_ level in cortex **(G)** and hippocampus **(H)** were examined with the ELISA assay. Representative fluorescent images of glial fibrillary acidic protein (GFAP; red), Aβ (green) and merged with 4′,6-diamidino-2-phenylindole DAPI; blue) for the cortex **(I)** and hippocampus** (J)** of the indicated mouse strains with three different treatments were depicted. The astrocytes marked by GFAP expression were morphologically activated in the transgenic mice but had no difference among three treatments. Scale bar: 100 μm. All data were analyzed with one-way ANOVA, Tukey’s multiple comparisons *post hoc*. *P < 0.5, **P < 0.01, ***P < 0.001.

The brain extracts well delayed the onset of Aβ-induced paralysis in worms. Simultaneously the oral administration of brain extracts containing Aβ or not in APP/PS1 mice were conducted to determine whether Aβ would be systemic transmissible. Six-month-old AD transgenic APP/PS1 mice and their WT counterparts were given brain extracts from WT or AD transgenic mice (14-month-old) via gavage for 14 days. Five months later, both WT and AD mice, receiving brain extracts or PBS control, were carefully evaluated. The main components of APP processing pathway were analyzed by Western blot in mouse forebrain of all six experimental groups. We found that the protein levels of APP and CTFs were markedly increased in the cortex and hippocampus of three AD groups; BACE1 protein levels were similar in all six groups (Figure 7). Moreover, total Tau (tTau), phosphorylated Tau (pTau) and the pTau/tTau ratio in the forebrain were not altered (Figures 7A–D).

By ELISA assay and Thioflavin-S staining, cerebral Aβ levels and Aβ deposition were determined. Aβ_1–42_ levels in AD mice receiving brain extracts did not differ from those receiving PBS, while in three WT groups, human Aβ_1–42_ could not be detected (Figures 7G,H). Similarly, Aβ deposits were equally obvious in the cortex and hippocampus of three AD groups while none was observed in three WT groups (Figures 7E,F).

As astrocyte- and microglial cell-associated chronic inflammation plays a detrimental role in AD progression (Ransohoff and Brown, 2012; Heneka et al., 2015; Ransohoff, 2016), we further investigated the changes of cerebral astrocytes and microglial cells using GFAP and Iba1 as markers respectively. GFAP positive astrocytes in three WT groups were all in the resting states, whereas astrocytic cells were activated and surrounding the Aβ deposits in the cortex and the hippocampus of three AD recipient groups, indicated by morphological changes as described in previous study (Heneka et al., 2015; Figures 7I,J). Similarly, activation of Iba1 positive microglial cells was also remarkable in the forebrain of three AD groups, but not in three WT groups (Supplementary Figure S5).

### Brain Extracts Do Not Alter the Behaviors of Mice

**Figure 8:**
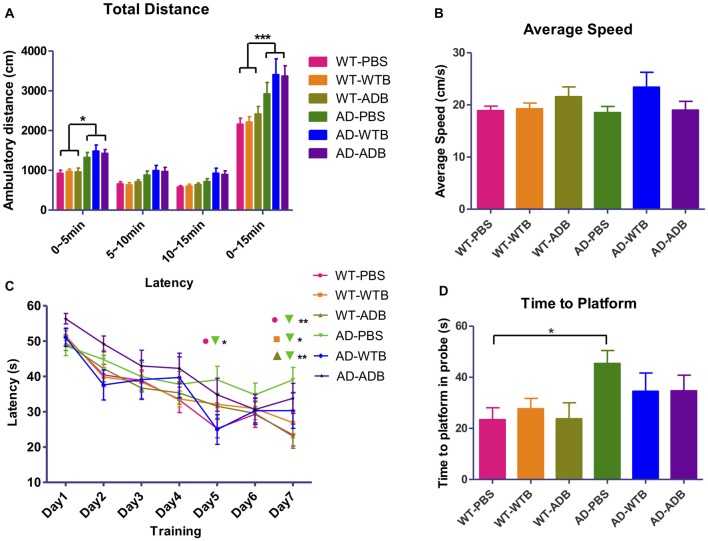
Feeding brain extracts at the age of 6 months did not alter the behaviors of mice. **(A)** The diagram summarizing the total ambulatory distance (cm) for each of the six groups (*n* = 9–22 per group) in the open-field test for the three 5 min blocks in 15 min observation. **(B)** The diagram representing the average swimming speed for different groups (*n* = 14–25) in Morris Water Maze test. **(C)** The average escape latency (four trails per day) for seven consecutive days and **(D)** Time for reaching the former platform location at the first time in the probe test. Diagrams in **(A,C)** were analyzed with two-way ANOVA, Tukey’s multiple comparisons *post hoc*. Diagrams in **(B,D)** were analyzed with One-way ANOVA, Tukey’s multiple comparisons *post hoc*. Significance level between certain groups are indicated with brackets (*P < 0.05, **P < 0.01, ***P < 0.001).

AD mouse models manifest neuropsychiatric and learning abnormalities during aging (Bonardi et al., 2011; Stover and Brown, 2012). Recipient mice with a 3-month incubation time in all the six experimental groups were evaluated in the open field test and the Morris water maze test. The open field test is used to assess locomotion and anxiety-like behavior. There was no obvious difference in total ambulatory distance among three WT groups or three AD groups in open field test, though the hyperactivity was detected in three groups of AD mice, which was indicated by their much longer ambulatory distance (Figure 8A). The Morris water maze has been widely utilized to assess spatial memory and learning. As the average speed did not significantly differ among all the experimental groups (Figure 8B), three WT groups showed better learning than AD mice receiving PBS on the 7th day of training. In probe test, AD mice receiving PBS took longer time to the location of former platform than WT mice receiving PBS (Figures 8C,D).

In addition, 3-month-old WT and AD mice were given brain extracts from old WT or AD transgenic mice (14-month-old) for 14 days. Mice with an 8-month incubation time after gavage treatment were sacrificed and protein expression related to APP processing pathway, Aβ load and glial activation were also screened. The expression of BACE1 protein in the cortex and hippocampus was close in the four experimental groups, while the APP and CTF levels in two AD groups was significantly increased compared to those in two WT groups (Supplementary Figure S6). The same as what we observed in 11-month-old mice with a 5-month incubation time after oral feeding, abundant Aβ deposits were detected in two AD mice groups while none were observed in two WT mice groups by Thioflavin S staining, and there was no difference between AD mice treated with WT or AD brain extracts (Supplementary Figure S7A). By GFAP staining, morphologically activated astrocytes surrounding Aβ were marked in the forebrain of AD mice receiving WT or AD brain extracts (Supplementary Figures S7B,C).

## Discussion

The APPswe/PSEN1DE9 (APP/PS1) strain is a widely studied AD mouse model. Previously, we and others had proved that APP/PS1 mice exhibited amyloid plaques as well as impaired long term potentiation (LTP) at 6-month-old, and the number of plaques in brain sections from 11 month to 13-month-old AD mice was much higher than in sections from younger mice (Hong et al., 2014). In this study, we chose 14-month-old AD mice and their counterparts as brain extract donors. Brain extracts were tested in mice and *C. elegans* simultaneously. Mouse brain extracts were supplied to liquid medium for culturing CL4176 and CL2006 AD transgenic nematodes. Aβ-induced paralysis was significantly delayed (Figures 1, 2). To exclude the effects of blood factors, perfused brain and peripheral tissue extracts were applied in the culture and we found that the suppression of paralysis was well maintained. Anti-paralysis effects of FBS and BSA were also demonstrated in CL4176 and CL2006. Both FBS and BSA at 1% concentration exhibited protective effects in CL2006. In CL4176 nematodes, FBS varying from 0.01% to 5% all showed protective effects, while BSA just showed minor effects on paralysis suppression at the lowest dose of 0.01%. The protective effects of FBS might be attributable to the nutrients, and growth factors (Eitan et al., 2015). BSA was shown to inhibit the formation of beta amyloid aggregates from monomeric peptide Aβ_1–40_ in a dose-dependent manner thus enhancing amyloid-beta activation of endothelial cells (Reyes Barcelo et al., 2009). Since less aggregation of Aβ might cause the increase of soluble oligomers of Aβ_1–40_, which was regarded as the most toxic formation of Aβ (Santos et al., 2012), the higher dose of BSA might inhibit the formation of fibrillar Aβ and promote the production of toxic Aβ oligomers. Consistently, only 0.01% BSA had protective effects in CL4176 nematodes. However, the growth promoting and precocious gonad development were observed only in CL4176 worms treated with brain and peripheral tissue extracts, but not in worms treated with FBS, BSA or caffeine (Figure 5). This indicated that tissue extracts and serums suppressed worm paralysis in different ways, and tissue extracts also varied from traditional chemicals which improved AD pathology, like caffeine. More or less surprisingly, serums from AD patients, even the serum from a very old patient (AD-18, 93 years old) exhibited similar anti-paralysis effects in CL4176 worms (Figure 6). All these results suggested that some natural components in mammalian tissues could mitigate Aβ-induced AD pathology.

By the treatment of BC, proteins in the brain extracts precipitated in a flocculent form by boiling, and most of the proteins were removed by centrifugation. Consequently, the anti-paralysis effects were compromised (Figure 3B), which suggests that proteins sensitive to heat was responsible for the suppression effects on paralysis in CL4176 nematodes. By PK treatment, most of proteins inside were disassembled into oligopeptides and single amino acids by proteinase K (Hilz et al., 1975; Müller et al., 1994), so that the components did not precipitate into the flocculent formation during boiling. Actually, no component was lost though the protein concentration declined. Instead, such treated extracts displayed enhanced capacities of paralysis suppression and growth promotion (Figure 3, Supplementary Figure S5). Zhang et al. (2016) reported that the bipeptide Tyr-Ala significantly prolonged the lifespan of WT *C. elegans* and extended the nematodes’ lifespan under heat/oxidative stress. Therefore, we proposed that PK-treated tissue extracts rich of oligopeptides were beneficial to worms indeed.

As to the mechanisms how brain extracts suppressed the worm paralysis, the transcriptome profiles of CL4176 nematodes offered some clues. The ubiquitin proteasome system, one of the main proteolytic systems that orchestrate protein degradation, is responsible for maintenance of the normal cellular physiology either through the degradation of proteins or through the elimination of damaged proteins including pathologic proteins like Aβ (Baptista et al., 2012; Papaevgeniou and Chondrogianni, 2014). Dysfunction of the TGF-β signaling in the brains of AD patients and in mouse models of AD may accelerate Aβ deposition and neurodegeneration (Tesseur et al., 2006) and silencing a subset of TGF-β signaling would promote adverse effects of AD in transgenic *C. elegans* including deposition of β-amyloid (Haque and Nazir, 2016). Here, MG132, an inhibitor of ubiquitin proteasome system (UPS), and SB431542, an inhibitor of TGF-β signaling pathway, both significantly diminished the effects of brain extracts in a dose-dependent manner. The suppressive effect of brain extracts on worm paralysis could be completely blocked by the inhibitor of TGF-β signaling pathway alone at a high dose (150 μM) or by combined inhibition of the TGF-β signaling pathway and the ubiquitin mediated proteolysis with low doses of inhibitors (50 μM), while low dose of SB431542 or MG132 alone just partially attenuated the effects of brain extracts. Since the upregulated genes in TGF-β signaling were also involved in the ubiquitin mediated proteolysis (*skr-14* and *skr-15*, Figure 4C), the molecules in each pathway possibly function across the networks. Thus, we propose that the high dose of SB431542 not only inhibited the TGF-β signaling but also interfered others and finally totally diminished the protective effects of brain extracts in AD transgenic nematodes. Our results suggested that elevated ubiquitin mediated proteolysis and TGF-β signaling both contributed to the paralysis-suppressive effect of brain extracts, coinciding with the protective roles of the two pathways in some AD pathological processes. The results also showed that cocktail drugs targeting TGF-β signaling pathway and ubiquitin mediated proteolysis could offer synergistic protective effects.

Interestingly, mTOR signaling pathway was also upregulated in worms with brain extract treatment. The mTOR signaling pathway has been found to be upregulated in AD patients and AD transgenic mouse models (An et al., 2003; Caccamo et al., 2010). Pharmacologically inhibiting mTOR signaling with rapamycin or genetic reduction of mTOR ameliorates AD-like cognitive and pathological deficits in AD mice by increasing autophagy (Caccamo et al., 2010, 2014). Plenty of evidences also show that reducing the activity of mTOR increases lifespan in different species including *C. elegans* (Richardson et al., 2015). In this study, enhanced mTOR signaling in brain extracts-treated AD transgenic nematodes is complex and needs further studies, as rapamycin alone delayed the paralysis progress, therefore exerted a protective role in AD pathology (Figure 4E). While we could not detect a synergistic phenomenon of rapamycin and brain extracts, this could be due to “ceiling effect” of the brain extracts.

Some natural factors, like tissue inhibitor of metalloproteinase 2 (TIMP2), is necessary for the cognitive benefits conferred by human cord plasma for targeting ageing- or disease-associated hippocampal dysfunction (Castellano et al., 2017). Growth differentiation factor 11 (GDF11) could slow the age-dependent deterioration of the neurogenic niche in mice and reverses age-related dysfunction in mouse skeletal muscle (Katsimpardi et al., 2014; Sinha et al., 2014). The brain extracts might also promote the production of endogenous growth factors and other active molecules, which help protect against the Aβ toxicity. Therefore, some natural factors inside the tissue extracts, or the expression of beneficial factors triggered by tissue extracts, might play important roles for the improvement of AD pathology in nematodes.

Meanwhile, brain extracts were orally administrated to APP/PS1 transgenic AD mice and their WT counterparts at the age of 3 and 6 months old. The intervention time were chosen because mice were in preclinical- and initiative- stage in the progression of AD, respectively. Human Aβ_1–42_ was not detected in the forebrain of 11-month-old recipient WT mice with a 5-month inoculation period by ELISA assay. Furthermore, cerebral β-amyloidosis was absent in wild type mice demonstrated by Thioflavin S staining (Figure 7). Similar results were obtained from 11-month-old recipient WT mice with an 8-month inoculation period (Supplementary Figure S7). The integrity of blood brain barrier impeded all the damaging molecules if there was any. In two cohorts of recipient AD mice, the expression of main components involved in APP processing did not alter (Figure 6 and Supplementary Figure S6). The oral treatment of AD brain extracts neither aggravated nor mitigated the Aβ load, glial activation (Figure 7, Supplementary Figure S7) and the abnormal behaviors (Figure 8). Notably particularly, the learning abilities of AD mice receiving brain extracts also did not differ from those of WT mice (Figure 8). Considering the extraordinary anti-paralysis effects of brain extracts in AD transgenic nematodes, the reasons why oral treatment of brain extracts in AD mice showed no effect on AD pathology were complicated. We could suppose the insufficient absorption of brain extracts, or inadequate dosage to take effect, or the treatment course not long enough, or the components fail to cross the blood brain barrier. Therefore, the effect of brain extracts in AD mice worth further researches with improved experimental methods.

Collectively, data from worms and mice in this study do not support the transmission of cerebral β-amyloidosis through oral administration of the Aβ-containing brain extracts. However, we found out the drastic effects of brain extracts on paralysis suppression in AD transgenic nematodes, which indicates that certain component(s) inside the brain extracts could ameliorate the AD pathology, partially through the ubiquitin proteasome system and the TGF-β signaling pathway.

## Author Contributions

FH, JF and LB: conceptualization and supervision. YY, MW, PY, JX and FH: methodology. YY: formal analysis. YY, MW, PY, ZW and LH: investigation. FH, JF, WW and MY: resources. FH and YY: writing—original draft, writing—review and editing. FH and JF: funding acquisition.

## Conflict of Interest Statement

The authors declare that the research was conducted in the absence of any commercial or financial relationships that could be construed as a potential conflict of interest.

## Abbreviations

Aβ: Beta-amyloid
AD: Alzheimer’s disease
APP23: Amyloid precursor protein carrying the Swedish mutation
APP/PS1: Amyloid precursor protein/presenilin 1
BACE1: β secretase
BC: boiling and centrifugation
BCA: Bicinchoninic acid
BSA: Albumin from bovine serum
*C. elegans*: *Caenorhabiditis elegans*
CJD: Creutzfeldt-Jakob disease
CTF: C-terminal fragments
DAPI: 4′,6-diamidino-2-phenylindole
DMSO: Dimethyl sulfoxide
GFAP: Glial fibrillary acidic protein
FBS: Fetal bovine serum
Iba1: Ionized calcium binding adapter molecule 1
LTP: Long term potentiation
mTOR: mammalian target of rapamycin
NGM: Nematode growth medium
NINCDS-ADRDA Alzheimer’s Criteria: Criteria proposed by the National Institute of Neurological and Communicative Disorders and Stroke and the AD and Related Disorders Association
PBS: Phosphate buffer saline
PK: treating with proteinase K and boiling
p-Tau: Phosphorylated tau protein
qRT-PCR: Quantitative real time Polymerase Chain Reaction
TGF-β: Transforming growth factor β
t-Tau: Total tau protein
UPS: Ubiquitin proteasome system
WT: wildtype.
