# Supplementary material for: The Aβ Containing Brain Extracts Having Different Effects in Alzheimer’s Disease Transgenic Caenorhabditis elegans and Mice
Source: Front Aging Neurosci. 2018 Jul 31;10:208. doi: 10.3389/fnagi.2018.00208 (PMC6079246; doi:10.3389/fnagi.2018.00208)
Supplement: Supplementary file 1 [file Presentation_1.pdf]

Supplementary Information

The Aβ containing brain extracts having different effects in Alzheimer's disease transgenic *Caenorhabditis elegans* and mice

Yufang Yang[1], Mo Wang[1], Ping Yang[3], Zishan Wang[1], Li Huang[1], Jing Xu[2], Wang Wei[5], Mei Yu[1], Liping Bu[4]*, Jian Fei[2]*, Fang Huang[1]*

Supplementary figures and legends

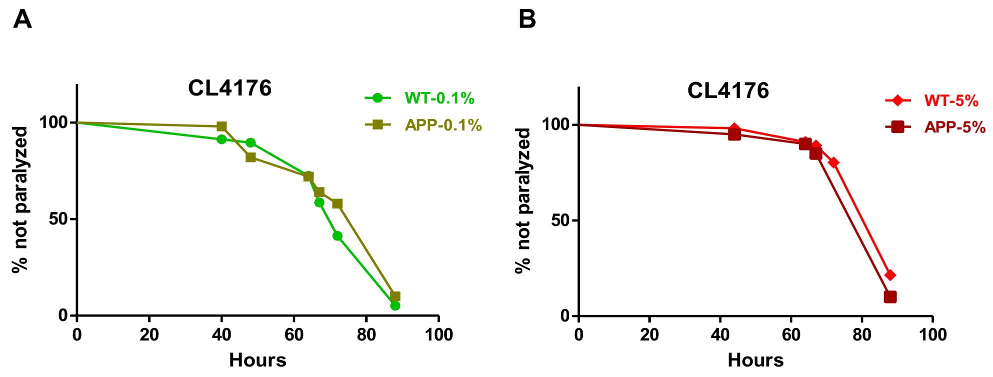

Figure S1. Comparable delaying effects of brain extracts from wild type mice and AD transgenic mice at both 0.1% and 5% concentration in AD transgenic nematode CL4176. (A) Effects of brain extracts from wild type mice in CL4176. **(B)** Effects of brain extracts from AD transgenic mice in CL4176. Data were analyzed using a paired log rank survival test.

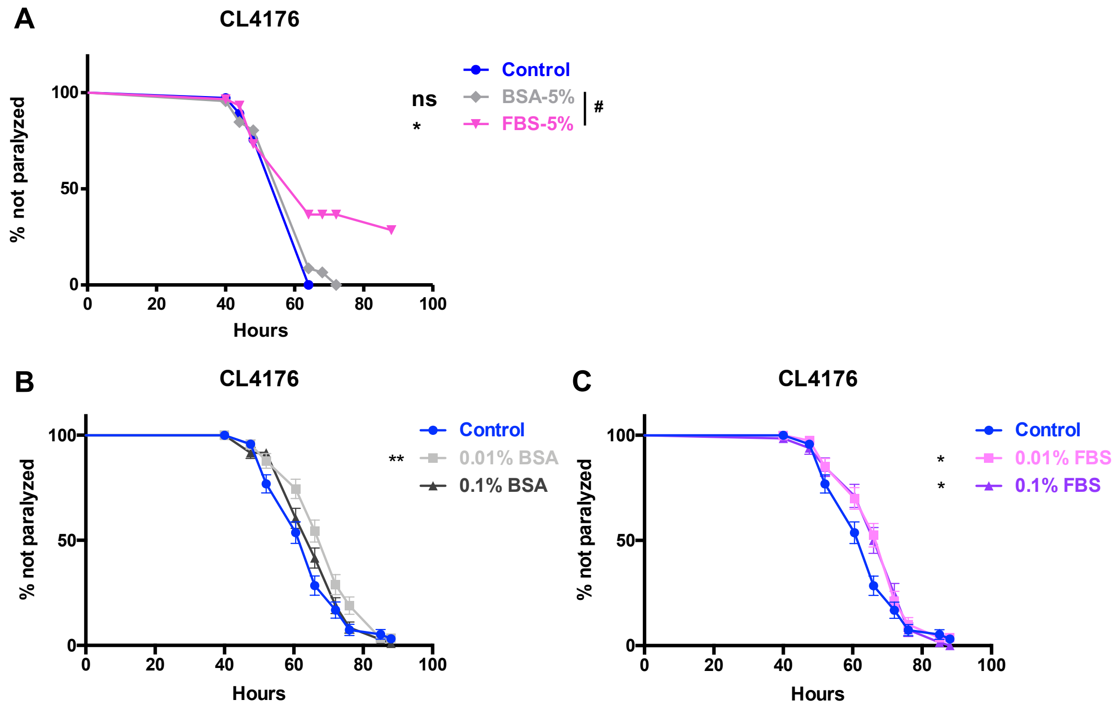

Figure S2. Effects of BSA and FBS on paralysis induced by Aβ expression in AD transgenic nematode CL4176. (A) Different effects of 5% BSA and 5% FBS on worm paralysis. **(B)** Effects of low dosage of BSA on worm paralysis. **(C)** Effects of FBS at low dosage on worm paralysis. Data were analyzed using a paired log rank survival test. The level

of significance was shown in brackets (* $P < 0.05$, ** $P < 0.01$, # $P < 0.05$, ns: $P > 0.05$, no significant difference).

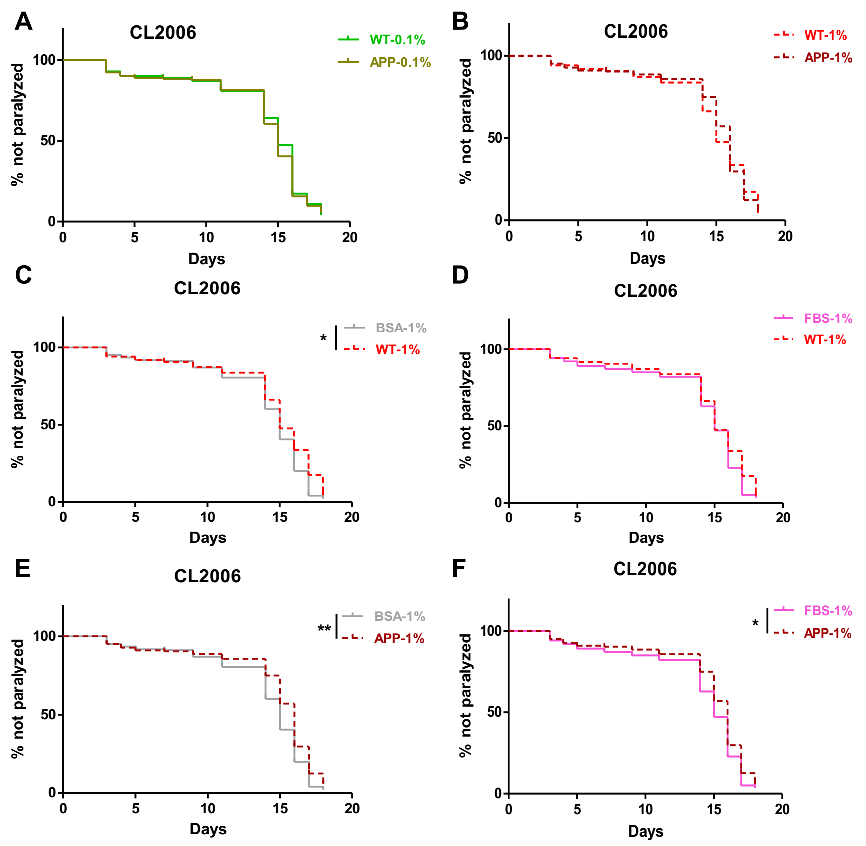

Figure S3. Comparisons among the effects of 1% BSA , 1% FBS and brain extracts from wild type mice and AD mice on paralysis in AD transgenic nematode CL2006. No difference was observed between the delaying effects of brain extracts from wild type mice **(A)** and AD transgenic mice **(B)** at both 0.1% and 1% concentration on AD transgenic nematode CL2006. **(C)** 1% BSA *vs* 1% brain extract of wild type mice. **(D)** 1% FBS *vs* 1% brain extract from wild type mice. **(E)** 1% BSA *vs* 1% brain extracts of AD transgenic mice. **(F)** 1% FBS *vs* 1% brain extract of AD transgenic mice. Data were analyzed using a paired log rank survival test. The level of significance was shown in brackets (* $P < 0.05$, ** $P < 0.01$).

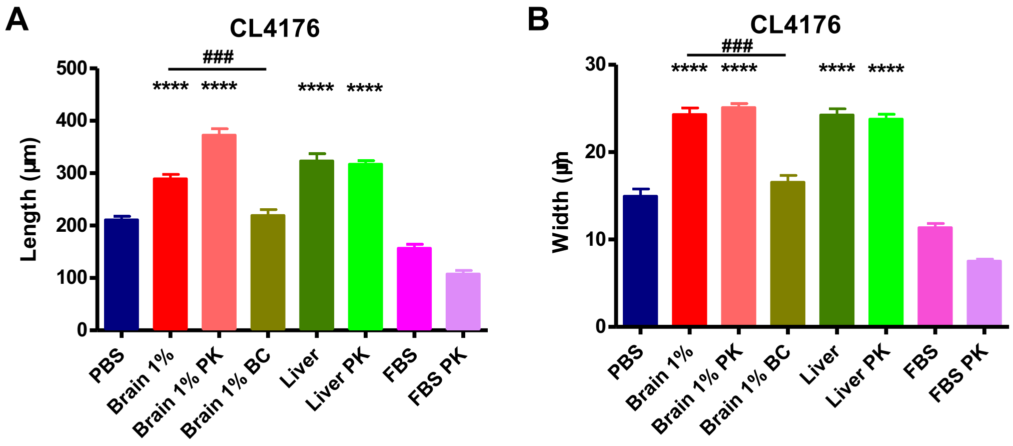

Figure S4. Similar effects on growth of CL4176 between feeding samples (brain extract, liver extract and FBS at 1% concentration) with PK treatment and their non-treated controls. (A) Lengths of worms collected at 88h after giving extracts. **(B)** Widths of worms collected at 88h after giving extracts. Data were analyzed using a paired log rank survival test. The level of significance was shown in brackets (**** $P < 0.0001$, ### $P<0.001$).

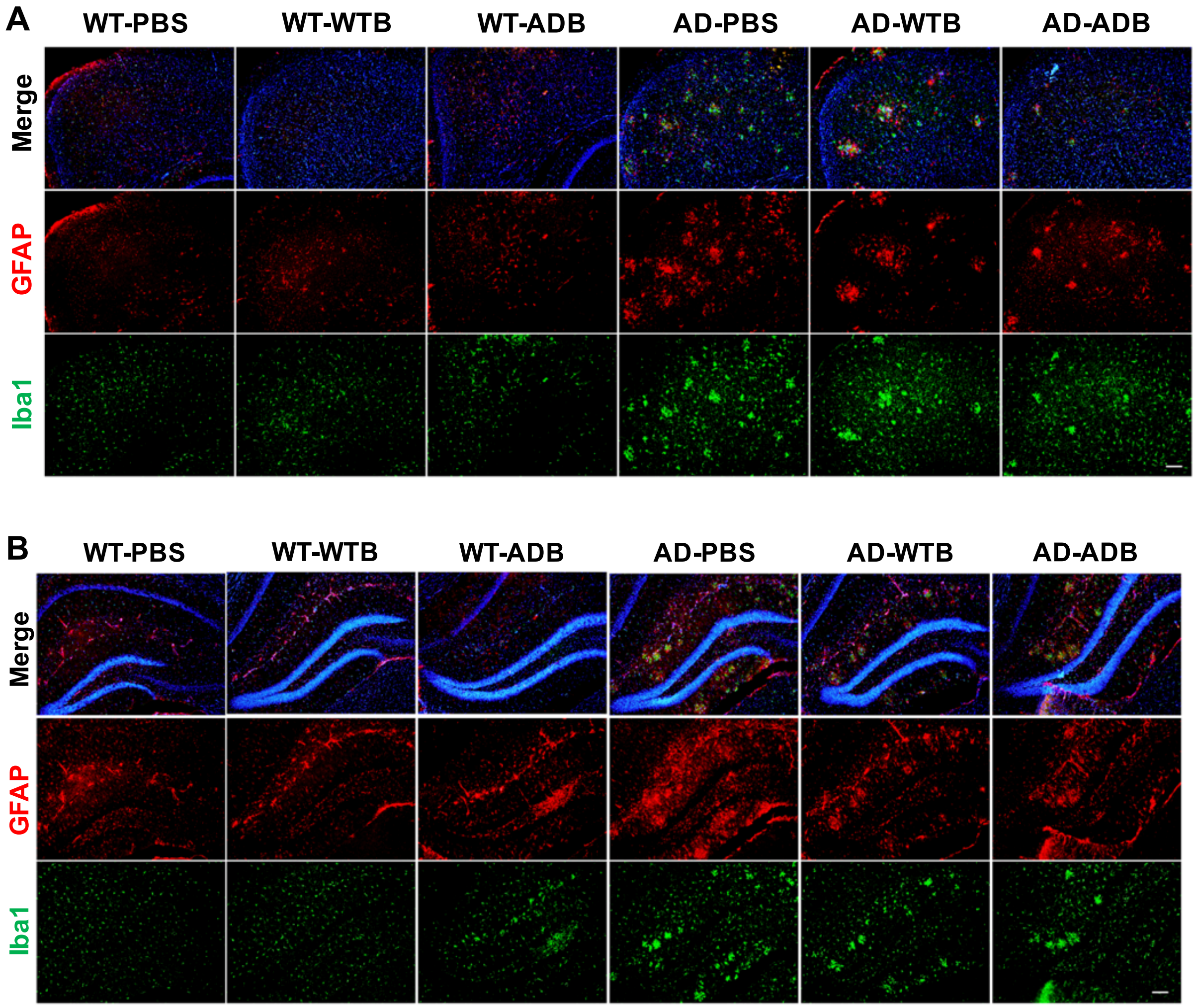

Figure S5. Astrocytes and microglial cells were activated in the brain of AD transgenic mice with a 5-month incubation time after gavage treatment at the age of 6 months old. Representative fluorescence images of GFAP (red), Iba1 (green) and merged with DAPI (blue) for the cortex **(A)** and hippocampus **(B)** of the indicated mouse strains with three different treatments are depicted. The astrocytes marked by GFAP and microglial cells marked by Iba1 were morphologically activated and were surrounding the plaques in the transgenic mice but have no distinguishable difference among three treatments. Scale bar: 100μm.

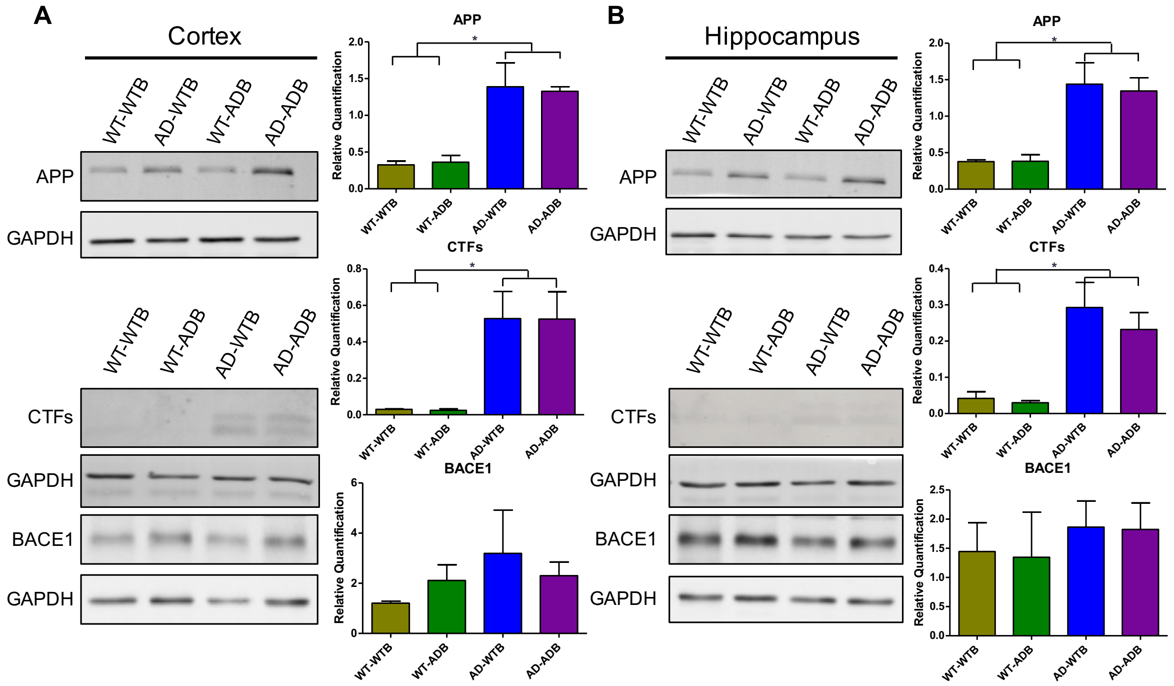

Figure S6. Protein levels of main components in Aβ generation pathway in the forebrain of mice with an 8-month incubation time after gavage treatment at the age of 3 months old. Representative western blot indicates the expression levels of APP, BACE1, and CTFs in the cortex **(A)** and hippocampus **(B)**. The relative expression levels of proteins were presented in the diagram. No significant difference was detected among three treatments in WT or AD mice (n = 3). GAPDH served as the internal control. All data were analyzed with one-way ANOVA, Turkey' s multiple comparisons post hoc, * $P < 0.05$.

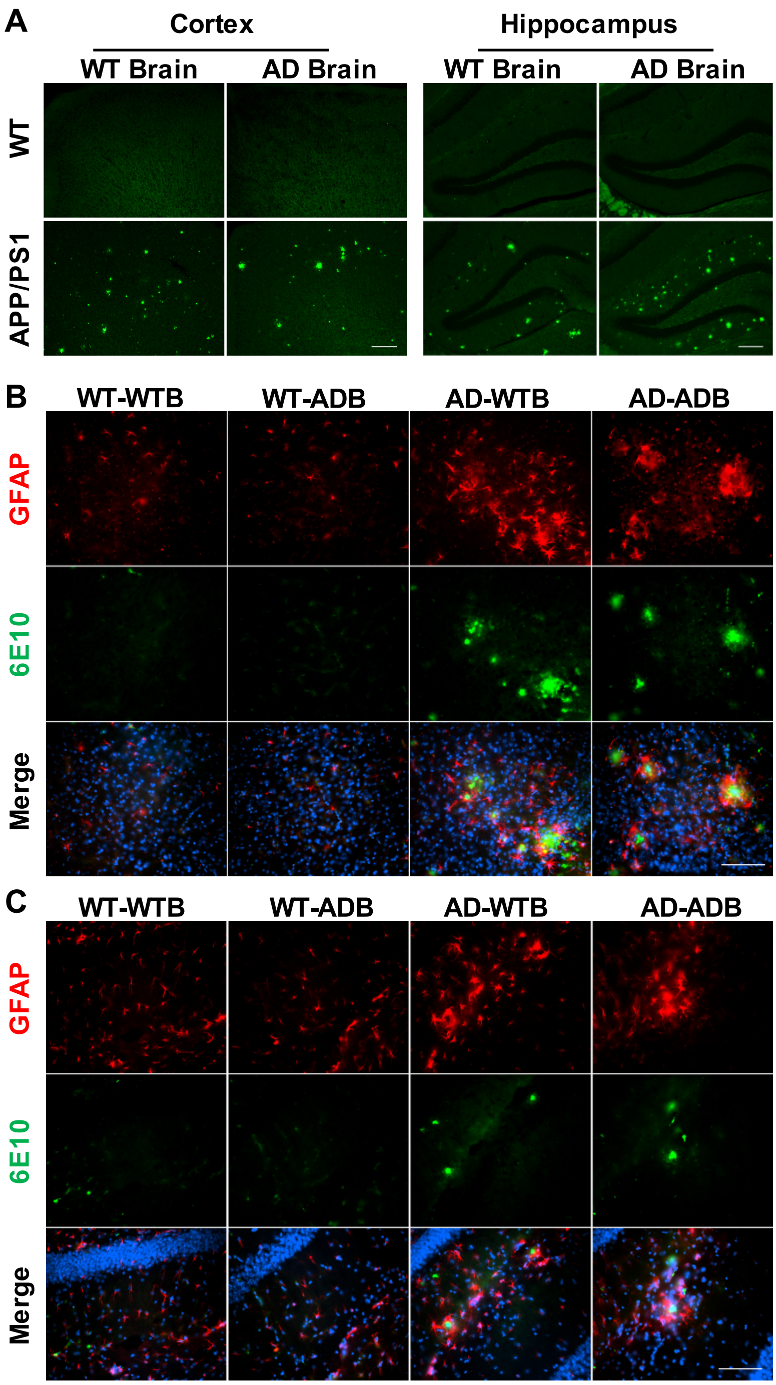

Figure S7. Aβ load and glial activation in the forebrain of 11-month-old AD transgenic mice with gavage treatment at the age of 3 months old. (A) Representative fluorescent images of Thioflavin-S staining sections from cortex and hippocampus. Astrocytes stained with GFAP (red) and β-amyloid stained with 6E10 (green) were merged with DAPI (blue) of the cortex **(B)** and hippocampus **(C)** of the indicated mouse strains with three different treatments. Scale bar: 100 μm.

Supplementary tables

Table S1. Primers of qRT-PCR for CL4176

Name	Sequence (5‘ to 3’)
Act-1-F	5’-CGAGCGTGGTTACTCTTTCAC-3’
Act-1-R	5’-GACTCCATACCCAAGAAGGATG-3’
skr-15-F	5’-GTCTAACGACAGAGTGGTCTTG-3’
skr-15-R	5’-GACTGTGTTACCAGATGGATAGG-3’
skr-17-F	5’-CAACCATCAGAGAGCTAGGATATG-3’
skr-17-R	5’-GTCCGGTAACGTCCAAATGA-3’
skr-14-F	5’- TCGCTGAATGGTGTGAGAAGC-3’
skr-14-R	5’- TTTGCCCTTTGCCATGTTGG -3’
ubc-9-F	5’-CCATTCGGATTCATTGCCAAAC-3’
ubc-9-R	5’-GGGTACACATTTGGATGGAAGAG-3’
rskn-1-F	5’-TGCAGTTATGGAGGAGAGAAAG-3’
rskn-1-R	5’-GATGGTGAGAGTGTCTCAGAAG-3’
ife-5-F	5’-GGTGGACGTTGGCTCATTAT-3’
ife-5-R	5’-CACGCAATTCCTATGTGTTTGG-3’

Table S2. Basic information AD patients and normal subjects.

Basic information of AD patients and normal subjects		
Patients	Serial No.	Range of Age
Normal	A5	20-24
Normal	A73	65-69
Normal	A8	65-69
Normal	A57	70-74
Normal	A35	21-24
Normal	A24	60-64
Normal	A91	60-64
Normal	A88	75-79
AD	64	55-59
AD	70	60-64
AD	66	65-69
AD	82	65-69
AD	18	70-74
AD	24	90-94
AD	60	60-64
AD	38	65-69
AD	53	65-69
AD	51	70-74
AD	84	95-99

Table S3. Protein concentration of tissue extracts by BC or PK treatment.

Protein concentration of extracts with BC or PK treatment				
Origin Samples	Origin concentration (mg/ml)	Treated samples	Final Concentration (mg/ml)	Depletion Percentage (%)
Brain	0.655	Brain 1% PK	0.29	55.25
Brain	0.655	Brain 1% BC	0.1	85.11
Liver	0.655	Liver 1% PK	0.35	46.74
FBS	0.655	FBS PK	0.51	21.54
All tissue samples are extracted from 2-month-old wild type mice.				
PK: Treated with Proteinase K at 58 ℃ for 2h then boiling for 10 min.				
BC: Boiling for 10 min and centrifuged at 3,000 g for 5 minutes.				
